# Using “Cinéma Vérité” (truthful cinema) to facilitate replication and accountability in psychological research^†^

**DOI:** 10.3389/fpsyg.2013.00872

**Published:** 2013-11-27

**Authors:** Jerry Suls

**Affiliations:** Behavioral Research Program, Division of Cancer Control and Population Sciences, National Cancer InstituteBethesda, MD, USA

**Keywords:** research practices, replication

## Abstract

To increase replication and accountability, it is proposed that researchers make audio/video recordings of laboratory protocols using currently available technologies, such as smart-phones. A detailed record of the procedure representing each experimental condition of the study design with simulated participants could then be posted on the internet and made accessible to researchers wanting more information about the procedures described in the research publication. Making recordings of all research participants a standard practice would be a greater challenge because of threats to internal validity and ethical concerns, however it is feasible and merits a broad discussion among researchers, professional societies, IRB’s and funding organizations.

The discovery of several high-profile cases of fraudulence in the psychological sciences has required the redaction of several articles published in prominent journals and has highlighted some problems in the research enterprise. Evidence has come to light of authors making-up data wholesale (e.g., Levelt, 2011) or selecting only statistically significant effects and treating them as the primary outcomes (Kerr, 1998; Simmons et al., 2011). In response, there have been calls for better oversight, greater transparency and more scientific replications (e.g., Giner-Sorolla, 2012; Koole and Lakens, 2012; Makel et al., 2012; Pashler and Harris, 2012).

The recent fraudulence cases helped to spotlight several long-standing problems associated with “normal” behavioral science practice, such as the rarity of replications. For example, a recent survey by Makel et al. (2012) reported an overall replication rate of 1.07% in psychology publications. This is a problem because replication not only helps to build a cumulative science, but also discourages fraudulent behavior. As Campbell (1979) observed, “Fields lacking the possibility or practice of competitive replication⋯lack an important social system supporting honesty,” (p. 258).

Remedies to reduce dishonesty (and also to maximize the use of existing data) have proposed to make use of the near-infinite storage capacity afforded by advances in computer technology, which can allow for data to be accessible to all (e.g., Nosek and Bar-Anan, 2012). Already, a plan for making data accessible is a requirement of research conducted with support exceeding $500,000 in any single year from the National Institutes of Health. In time, making all data publicly accessible may become a common practice. Open access to data may provide a deterrent to bad practices, encourage the merging of data sets and allow the data to be maximally utilized to answer questions that had not originally occurred to the original researchers.

A complementary strategy is proposed here to facilitate replication and transparency. Thus far, most responses to the current crisis have focused on the “dependent variable” aspects of the research enterprise (Adolph et al., 2012 is an exception), but the independent variable/research protocol side also requires more attention. All published research reports provide some information about design and procedures, but the degree of detail ranges widely with the subfield and the journal. In some instances, the reader has access to measures such as questionnaire items. (These, too, can be made publicly available along with the data.) Some perception and cognition paradigms, particularly those using computer-based protocols/software, sometimes are made available as online supplementary material to the publication. But these are the exceptions and not the rule in psychological science.

In certain fields of psychology (such as personality and social psychology), study authors however, rarely have the luxury to provide extensive information about protocol presentation – the setting, experimental script, timing and delivery of the materials or independent variables. Also, some aspects of the setting or delivery probably cannot be adequately verbalized, even if journal space permitted. To the degree that certain kinds of studies involve “stage management” to achieve mundane and experimental realism (see Aronson et al., 1998), other researchers considering a replication are left to their imaginations.^1^ Even a lengthy correspondence or conversation with the investigators of the published report, albeit informative, probably leaves many details unknown. These small, sometimes subtle, things are not supposed to matter, but experienced researchers often learn, after the fact, that they do.

This lack of complete procedural information is a barrier to conduct direct replications. To be clear, a *direct replication* refers to repetition of an experimental procedure, in contrast to “repetition of a test of a hypothesis or a result of earlier research work with different methods,” which is a conceptual replication (Schmidt, 2009). Whether the lack of procedural information is the most serious barrier to (direct or constructive) replication is complicated by replication tending to be perceived as lacking prestige and originality. Further, both editors and reviewers, at least prior to the latest fraud cases, have not been sanguine about publishing replications (Lyndsay and Ehrenberg, 1993; Neuliep and Crandall, 1993; see Suls and Martin, 2009).

Even if replication was not the goal, complete details about procedural protocols are essential for evaluating and using open access data, which are becoming more popular, if not required. Merging of distinct data sets or conducting secondary analyzes scarcely make sense if one does not know exactly what procedures were used by the original investigators.

In the past, there were no readily available technologies to collect information about the subtleties of procedure delivery, but that is no longer the case because of advances and availability of recording devices. Filming research procedures on a digital camera or smartphone is relatively simple and straightforward. Then these audio-video snips could be posted in a “You-Tube library of psychological research procedures” (or available as supplementary material linked to the published report). Of course, to record the subtleties of procedural delivery it may be necessary to use more sophisticated tools than smart-phones, but advances in audio/video recording technology, along with lower costs, can make this feasible for the vast majority of researchers.

Something similar has already been proposed by Adolph et al. (2012) in the context of development research, which often uses audio and video-recordings as its primary data (see  and openshapa.org). While Adolph et al. (2012) believe making raw data recordings available to others would increase transparency, their primary goal is to facilitate data sharing. To be clear, recordings used in developmental science are somewhat unique because they typically include both the research procedures *and* the data (i.e., the child’s responses) because both the procedures and resulting behavior unfold over the laboratory session. In fact, the particular type and sequence of research procedures may be contingent upon participant’s prior behaviors. In other areas of research, however, procedures are followed independently of how the participant responds. This is important because, for the purpose of mounting a direct replication, only recording of the procedures may be needed.

Some questions and concerns can be anticipated about using cinéma vérité for research purposes. Does the proposal mean that all participants in a research study will be recorded? If this were feasible, it would serve the triple missions of aiding replication, data sharing and transparency. These laudatory goals are what Adolph et al. (2012) envision for the research enterprise. There are some downsides and challenges, however. Research participants would have to provide informed consent to being filmed and potentially identifiable. If consent to being filmed occurred prior to the experimental session, participant reactivity might increase, thereby affecting the internal validity of the experiment. (In contrast to adult participants, in developmental protocols with young children, parents provide the consent and the children may be insensitive to the camera’s and therefore less of a problem.) Also, filming all participant sessions would require extra IRB hurdles that might encumber the research project. Further, there might be such a thing as “too much data.” Recording each participant’s laboratory/field session might be overkill. The cost-benefit ratio associated with making audio–video records of all research sessions is difficult to judge in the absence of concrete examples.

The challenges to filming actual research participants might seem insurmountable because of threats to privacy and internal validity. However, both participants and IRB’s might be amenable if assurances are provided. You-Tube and similar applications, for example, permit “auto-blurring” so the person can be visually unidentifiable and audio-editing can distort a participant’s voice. Although participants’ reactivity to knowing they are being recorded might pose a threat to internal validity, IRB’s might be amenable to hidden recorders during the experimental session as long as participants have the right to refuse to release the material when the session has concluded and the participant has been debriefed. There is some precedent for this as many labs currently seek participant permission to show video excerpts in research contexts such as scientific meetings (Adolph et al., 2012).

## RECORDING SIMULATED PARTICIPANTS STILL HAS BENEFITS

Even if the hurdles are too great for recording real subjects, having recordings of the protocol delivery for each condition of an experiment with *simulated participants* and the laboratory context in which it occurs would be of substantial value as an archive for scholars, students and future researchers – both to evaluate the research findings and/or to conduct a replication. This proposal need not be restricted to laboratory experiments. Video/audio recordings of field experiment protocols, which typically involve special “stage management and direction,” might be of value to assess the value of the original research findings and provide sufficient detail to future researchers for replication.

Past practice has assumed that trivial differences in lab contexts (e.g., room size, lighting, temperature, configuration, experimenter’s tone of voice or posture, etc.) should not affect results in any meaningful way, especially if the theory or idea being tested seems to have nothing to do with context or delivery. Such assumptions have two consequences: (a) they encumber strict replication because future researchers do not possess a full knowledge about the original protocol and (b) potentially obfuscate whether seemingly “small” or “trivial” changes, in combination or independently of the manipulated factors, contributed to the results. As McGuire (1969) observed, “It is a wise experimenter who knows his artifact from his main effect; and wiser still is the researchers who realizes that today’s artifact may be tomorrow’s independent variable,” (p.~13). As an example, the substantive empirical literature on the self-fulfilling prophecy (Snyder and Haugen, 1995), still a thriving area of social psychology, was inspired by demonstrations of the unintended effects of experimenter expectations on research participants’ behavior in the laboratory (Rosenthal, 1966).

In summary, letting the cameras roll, or “Cinéma Vérité” (truthful cinema) – a French film movement of the 1950s, is proposed as an addition to behavioral research practice in the interests of replication and transparency. Although recording of simulated participants can aid the replication movement now, making raw procedure-data recording recording universal will require extensive discussions among professional scientific societies, academic institutions, IRB’s and funding sources rather than depend on lone investigators to grapple with their IRB’s. The technology is available; in fact, a large portion of the population carries one gizmo or another that makes recording simple and straightforward. Smart phones are carried in subways, shopping malls, classrooms, etc. Perhaps they also belong in the research laboratory. Film has been described as being able to see more than “meets the eye.” Perhaps film can also afford researchers the opportunity to see (and hear) more than the bare-bones descriptions that typically appear in scientific journals.

## Conflict of Interest Statement

The author declares that the research was conducted in the absence of any commercial or financial relationships that could be construed as a potential conflict of interest.

## Footnotes

1 Several years ago, a graduate student told me about her experience when a famous researcher visited her department to give a colloquium. She approached him explaining she was having a terrible time replicating one of the classic phenomena he discovered and asked whether they might talk for a while. He graciously agreed and after she described the procedures she used, the eminent researcher admitted they seemed entirely comparable to his own. However, he obtained the effect, but the graduate student had not, despite repeated attempts. Together they puzzled about this for some time without resolution. Finally the eminent researcher said, ``Well, I guess some of us know how to do it and some of us don't.'' At which point, the meeting ended.
